# Extracellular vesicles in bovine reproduction: their journey from gametogenesis to pregnancy

**DOI:** 10.3389/fcell.2026.1846335

**Published:** 2026-06-09

**Authors:** Yulia N. Cajas, Rosane Mazzarella, Meriem Hamdi, Gemma Millán de la Blanca, Juliano Coelho da Silveira, Claudia Lima Verde-Leal, Encina González, Dimitrios Rizos, Karina Cañón-Beltrán

**Affiliations:** 1 Department of Animal Reproduction, National Institute for Agriculture and Food Research and Technology (INIA-CSIC), Madrid, Spain; 2 Department of Biological Science, Technical University of Loja (UTPL), Loja, Ecuador; 3 Institute of Veterinary Anatomy, Vetsuisse Faculty Zurich, University of Zurich, Lindau, Switzerland; 4 Departamento de Medicina Veterinária, Faculdade de Zootecnia e Engenharia de Alimentos, Universidade de São Paulo (FZEA-USP), Pirassununga, Brazil; 5 Department of Anatomy and Embryology, Veterinary Faculty, Complutense University of Madrid (UCM), Madrid, Spain; 6 Escuela de Ciencias Agrícolas y Ambientales, Pontificia Universidad Católica del Ecuador, Ibarra, Ecuador

**Keywords:** cattle, embryo development, embryo maternal-communication, extracellular vesicles, miRNAs, oviduct, uterus

## Abstract

Extracellular vesicles (EVs), including exosomes and microvesicles, play a pivotal role in bovine reproduction by mediating communication between gametes, the developing embryo, and the maternal reproductive tract. These vesicles act as carriers of bioactive molecules, such as microRNAs, mRNAs, lipids, and proteins that regulate processes from gametogenesis to early pregnancy. The embryo-maternal dialogue, mediated by EVs, shapes the oviductal and uterine microenvironments, ensuring proper fertilization, embryonic development, and implantation. However, *in vitro* systems often lack the complexity of *in vivo* interactions, resulting in reduced embryo quality and developmental competence. This review summarizes recent findings on EV-mediated mechanisms of intercellular communication during the pre-implantation stages and discusses current challenges and emerging opportunities in developing *in vitro* models that more closely replicate physiological conditions within the bovine reproductive tract. Furthermore, it highlights the potential of EVs as diagnostic and therapeutic tools for improving reproductive efficiency and outcomes in cattle.

## 1 Introduction

Extracellular vesicles (EVs) are lipid bilayer-enclosed nanoparticles secreted by virtually all cell types and have emerged as mediators of intercellular communication (Almiñana and Bauersachs, 2020). They are commonly classified by size into small EVs (<200 nm) and large EVs (>200 nm), and by biogenesis into exosomes (30–100 nm), microvesicles (100–1,000 nm) and apoptotic bodies (50–4,000 nm), with additional subtypes such as prostasomes (Andrews et al., 2015) and uterosomes (Griffiths et al., 2008a), according to their tissue of origin. However, these classification frameworks often overlap and remain operational rather than definitive, as current isolation methods do not consistently distinguish EVs subpopulations with high specificity. This heterogeneity is reflected in their cargo composition, which includes proteins, lipids, and multiple RNA species, and is influenced by both cell of origin and physiological context (Cocucci and Meldolesi, 2015; Théry et al., 2018; van Niel et al., 2018; Almiñana and Bauersachs, 2020). Importantly, variability in EVs isolation, characterization, and analytical approaches can substantially affect reported cargo profiles and downstream functional interpretations.

**FIGURE 1:**
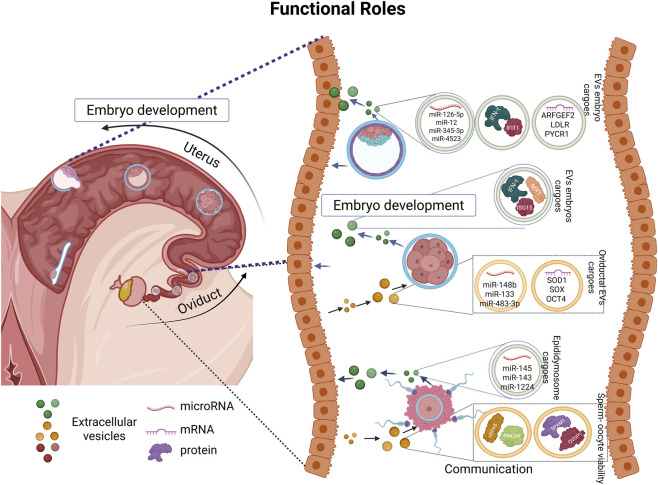
Schematic representation of gamete or embryo-maternal cross-talk in the reproductive tract mediated by EVs secreted by oocytes, spermatozoa, embryos, and the oviductal and endometrial epithelium. EVs play a crucial role in paternal and maternal–embryonic intercellular communication. They contribute to sperm and oocyte viability, regulate the expression of genes related to embryo quality, and modulate specific miRNAs associated with proliferation, differentiation, and survival during early embryonic development. Additionally, EVs enhance blastocyst quality and reduce oxidative stress. Embryos uptake these EVs, which regulate different signaling pathways. Similarly, embryo-secreted EVs contain proteins, miRNAs, and mRNAs that influence local responses in the oviduct and uterus. Elements are schematic and not drawn to scale. Figure created with BioRender.com.

In the reproductive tract, EVs have been associated with processes such as gamete maturation, fertilization, embryo development, and embryo-maternal communication (Figure 1). While these roles are increasingly reported, much of the supporting evidence derives from *in vitro* systems or molecular profiling studies and therefore should be interpreted with caution when extrapolating to *in vivo* physiology. Their ability to modulate the oviductal and uterine microenvironments has positioned them as potential candidates for diagnostic and therapeutic applications in reproductive biotechnologies (Supplementary Table S1), although their practical implementation remains limited by methodological and biological variability.

In cattle, EVs secreted by embryos and surrounding tissues have been associated with embryo quality and developmental competence. Mellisho et al. (2017), Mellisho et al. (2019) reported that EVs supplementation can improve *in vitro* embryo development and quality, while miRNA profiling studies of EVs from follicular fluid (FF) and embryo culture media have identified candidate biomarkers for oocyte competence and embryo viability (Gebremedhn et al., 2020). Follicular-fluid EVs (FF-EVs) have been proposed to mediate communication between somatic follicular cells and the oocyte, potentially supporting follicular growth and oocyte maturation. Functionally relevant molecules identified in FF-EVs include proteasome-related proteins (e.g., PSMA1, PSMC6) and miRNAs such as bta-miR-615, which targets *IGF2* (Mendonça et al., 2015; da Silveira et al., 2017). However, the functional contribution of these molecules when delivered via EVs, as opposed to other regulatory pathways, remains insufficiently characterized.

Sperm maturation is also linked to EV-mediated communication within the epididymal lumen. Epididymosomes, secreted by epididymal epithelial cells, transfer proteins and miRNAs implicated in the acquisition of sperm motility and fertilizing capacity (Sullivan, 2015). These EVs have been associated with remodeling of the sperm proteome and membrane lipid composition, processes required for capacitation and acrosome reaction. During fertilization, oviductal fluid-derived EVs interact with sperm and have been reported to influence calcium signaling and protein phosphorylation (Franchi et al., 2020). Despite these observations, the EVs-mediated modulation of sperm function remains incompletely understood.

Oviductal and uterine EVs (UF-EVs) contain bioactive molecules such as mRNA, miRNA, lipids and metabolites that have been associated with embryonic gene expression, metabolism, oxidative stress response, and overall developmental competence (Almiñana et al., 2017; Gatien et al., 2019; Leal et al., 2022) (Figure 1). These data support the concept of embryo–maternal communication; however, they predominantly reflect associative patterns rather than direct mechanistic evidence. Embryos have been shown to both secrete and internalize EVs, suggesting a potential feedback mechanism. In line with this, supplementation of uterine EVs during *in vitro* culture has been reported to enhances blastocyst formation, reduce apoptosis (↓Bax), and modulate pluripotency markers (↑Oct4), thereby improving implantation potential.

Overall, while EVs are increasingly recognized as important components of reproductive biology, the field is characterized by substantial methodological variability and a predominance of descriptive and *vitro*-based studies. Given the methodological variability in EVs research, a comparative assessment of key studies and their compliance with MISEV guidelines is provided (Supplementary Table S2). This review aims to critically evaluate the current evidence on EVs and their biomolecular cargoes throughout bovine reproduction from gametogenesis to early embryonic development and pregnancy establishment with emphasis placed on distinguishing between correlative and functional evidence, as well as highlighting methodological techniques. Figure 1 provides a schematic overview of the origins, types, and biological functions of EVs across key reproductive stages.

## 2 The role of EVs in oocyte maturation

**FIGURE 2:**
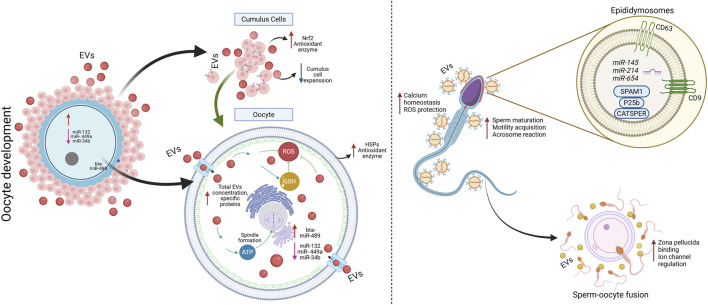
Extracellular vesicle (EVs)-mediated communication in reproductive processes. EVs from granulosa cells, oviductal epithelium, and epididymis carry miRNAs, proteins, and lipids that regulate oocyte maturation, sperm remodeling, and embryo development. Epididymosomes mediate sperm maturation by delivering proteins and small RNAs, with region-specific cargo contributing to sperm remodeling and preparation for fertilization. EV cargo is dynamic, responding to hormonal, environmental, and stress conditions to modulate gamete function and developmental competence. Elements are schematic and not drawn to scale. Figure created with BioRender.com.

The ovarian follicle is a highly specialized microenvironment composed of the oocyte, surrounded by granulosa (GCs) and cumulus cells (CCs), and enclosed by a theca cell layer that is separated by a basement membrane. During folliculogenesis, formation of the antral cavity results from coordinated hydrostatic pressure, osmotic gradients, epithelial fluid transport, and directional secretion, leading to the accumulation of FF primarily derived from thecal vasculature (Rodgers and Irving-Rodgers, 2010). Beyond its structural role, FF provides a biochemical milieu that supports bidirectional communication between the oocyte and surrounding somatic cells through paracrine signaling, gap junctions, and EVs, which transport bioactive molecules such as proteins, lipids, mRNAs, and miRNAs that influence oocyte competence and follicular dynamics (da Silveira et al., 2015; Andrade et al., 2019; de Ávila et al., 2020) (Figure 2). However, much of this evidence remains associative, and the extent to which EV cargo directly modulates oocyte developmental outcomes *in vivo* is still incompletely defined.

A key unresolved question concerns the mechanism by which oocytes internalize these EVs, given physical barriers such as the zona pellucida and the follicular antrum. In this context, specialized structures called transzonal projections (TZPs) which extend from CCs through the zona pellucida to the oocyte membrane have been proposed as conduits for selective EV uptake. Recent evidence provides direct mechanistic support for EVs-mediated communication within the follicular environment, demonstrating that FF-derived small EVs are internalized by CCs and transported through TZPs into the perivitelline space and ooplasm. Notably, EVs uptake is enhanced under metabolic stress conditions, and their supplementation during *in vitro* maturation improves oocyte nuclear maturation, blastocyst quality, and lipid metabolic profiles, suggesting a supportive role of EVs in preserving oocyte competence under adverse conditions (Lipinska et al., 2025). These mechanistic uncertainties complicate interpretation of functional studies, particularly those performed under *in vitro* conditions where EV concentrations and exposure routes may not reflect physiological settings.

The first description of EVs in FF was reported in mares in 2012 (da Silveira et al., 2012), revealing that granulosa cells can internalize FF-EVs both *in vivo* and *in vitro*. Notably, the miRNA content in FF-EVs varies according to donor age and oocyte quality, suggesting that EVs may reflect the physiological status of the follicle (da Silveira et al., 2012). In cattle, miRNAs found in granulosa cells- derived EVs regulate pathways such as PI3K-Akt, MAPK, and WNT, which are essential for oocyte maturation and follicular development (Andrade et al., 2019) (Figure 2). Nevertheless, these associations are largely based on bioinformatic predictions or transcriptomic correlations, and direct functional validation of specific EVs cargo molecules remain scarce.

Functionally, several studies report that FF-EVs influence both somatic cell behavior and oocyte developmental potential. For example, EVs from small (3–5 mm) and large (9 mm) bovine follicles promote CCs expansion and GCs proliferation in a size-dependent manner (Hung et al., 2017), while supplementation of EVs during *in vitro* maturation (IVM) can increase blastocyst yield and alter transcript levels, as well as DNA hydroxymethylation and methylation patterns in embryos (da Silveira et al., 2017), indicating a role in epigenetic regulation during early development. Although these findings support the modulatory role of EVs, they are predominantly derived from *in vitro* supplementation experiments, which are highly sensitive to methodological variables such as EV isolation techniques, dosage, and culture conditions. Consequently, extrapolation to *in vivo* physiology should be made with caution.

Similarly, EV supplementation has been associated with improved oocyte cryotolerance following vitrification. Oocytes exposed to these EVs exhibited preserved spindle integrity, reduced DNA fragmentation, and increased blastocyst development, expansion, and hatching rates compared with vitrified controls, highlighting the protective role of EVs against vitrification-induced damage (Diaz-Muñoz et al., 2025).

Importantly, EVs signaling is not static but adapts to environmental and physiological cues. For instance, EVs derived from heat stressed animals or follicles of different sizes display distinct molecular profiles and have been reported to mitigate stress-induced damage during IVM (Andrade et al., 2019; Morales Dalanezi et al., 2019; Ying et al., 2023), while oxidative stress induces GCs to secrete EVs enriched in antioxidants that protect neighboring cells (Saeed-Zidane et al., 2017). This suggests an adaptive role for EVs-mediated signaling, but most studies rely on differential expression analyses, and causal links between specific EVs cargo components and functional outcomes remain largely unexplored. Recent findings indicate that, although FF-derived EVs supplementation may not significantly affect nuclear maturation or embryo development rates, but instead modulates metabolic parameters such as lipid metabolism and mitochondrial activity (Schneberger et al., 2026). This apparent disconnect between metabolic changes and developmental endpoints underscores the complexity of interpreting EVs function and suggests that commonly used metrics may not fully capture oocyte competence. It also reinforces the importance of methodological standardization, as differences in EVs isolation, quantification, and experimental design can substantially influence reported outcomes.

**FIGURE 3:**
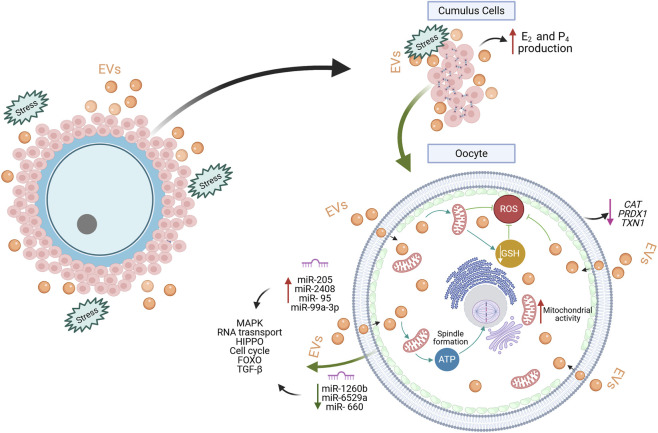
Schematic representation of the oocyte and surrounding cumulus cells illustrating the bidirectional exchange of EVs. Progesterone (P4) levels regulate the miRNA cargo of FF-EVs, with low P4 conditions associated with enrichment of miRNAs involved in oocyte maturation, RNA processing, and translation. As P4 increases, FF-EVs deliver bioactive molecules to oocyte and cumulus cells, modulating gene expression and altering transcripts related to cell cycle progression and differentiation during *in vitro* maturation (IVM). Elements are schematic and not drawn to scale. Figure created with BioRender.com.

Therefore, these studies demonstrated that EVs are dynamically modified according to their follicular origins and stimuli, playing a key role in modulation of the follicular environment. Research on the content and effects of EVs from different follicular environments is needed for a better understanding their roles in follicular physiology. Recent studies further underscore the signal specificity of FF-EVs. For example, FF-EVs isolated from different stages of the estrous cycle exposed to low and high progesterone (P4) levels displayed 161 differentially expressed miRNAs, all upregulated in the low P4 conditions (de Ávila et al., 2020) (Figure 3). These miRNAs were associated with oocyte maturation, translation and RNA processing pathways, and when supplemented during IVM, significantly altered transcripts involved in cell cycle regulation and differentiation in COCs (de Ávila et al., 2020). Moreover, body energy reserves (Bastos et al., 2023) and seasonal temperature variations (Gad et al., 2023) can influence the miRNA composition of FF-EVs, highlighting their sensitivity to systemic metabolic and environmental factors.

Beyond the follicle, FF-EVs have been detected in the oviduct following ovulation, where they can modulate gene expression in oviductal epithelial cells and potentially influence fertilization and early embryo development (Almiñana et al., 2017; Almiñana and Bauersachs, 2020; Fang et al., 2022; Bastos et al., 2023; Liao et al., 2025; Chaves et al., 2026). However, the extent to which these effects occur under physiological conditions, and their relative contribution compared to other signaling mechanisms, remains to be determined.

In conclusion, EVs are increasingly recognized as context dependent mediators of communication within the follicular environment, with potential roles in coordinating somatic cell function and oocyte competence. Nevertheless, the field is currently limited by significant methodological variability, incomplete mechanistic understanding, and a predominance of *in vitro* and correlative evidence. Future studies should aim to map EVs content across follicular stages and stress conditions and elucidate the mechanisms of EVs uptake and cargo delivery to oocytes to fully exploit their potential in reproductive technologies.

### 2.1 Follicular Fluid-EVs: molecular cargo and emerging biomarker potential

The FF represents a valuable model for studying EVs- mediated signaling, as both the source and recipient cells are relatively well-defined, and RNA molecules remain stable within this microenvironment (Gebremedhn et al., 2020). Experimental evidence suggests that FF-EVs can elicit distinct transcriptomic responses in bovine oviduct epithelial cells (BOEC) compared to whole FF, particularly during short-term *in vitro* exposure (Tesfaye et al., 2021). While these findings support a role for EVs as mediators of intercellular communication, they are primarily based on *in vitro* systems, and their physiological relevance remains to be validated *in vivo*.

The proteins and RNA cargo of FF-EVs, has been extensively characterized (da Silveira et al., 2012). Several of these molecules are associated with key reproductive processes such as oocyte competence, embryo development and follicular signaling pathways (Uzbekova et al., 2020; Rodrigues et al., 2019). For instance, proteasome subunit proteins (PSMA1, PSMA5, PSMA6, PSMC6, PSMD2) identified in bovine FF-EVs are implicated in protein turnover, oxidative stress response, and cell cycle regulation (Tanaka, 2009). Similarly, specific miRNAs present in FF-EVs, such as bta-miR-615 and bta-miR-323, have been linked to pathways involved in follicular growth, meiosis resumption, steroidogenesis and early embryonic development through predicted targeting of genes including IGF2 (Mendonça et al., 2015) and have been proposed to regulate genes involved in embryo development and cell differentiation (da Silveira et al., 2017; Zhang et al., 2013). However, despite this growing body of descriptive data, the classification of FF-EVs cargo as *bona fide* biomarkers remains premature. According to the classical definition, biomarkers must be measurable indicators with demonstrated diagnostic, prognostic, or predictive value. In the case of FF-EVs, most studies report associations between specific molecules and reproductive outcomes, but lack robust validation of their sensitivity, specificity, and reproducibility across independent cohorts.

Furthermore, the identification of candidate biomarkers is highly influenced by methodological variability. Differences in EVs isolation techniques, such as ultracentrifugation (UC) versus size-exclusion chromatography (SEC), for example, can produce vesicle populations with distinct compositions and biological activities, thereby affecting downstream analyses and biomarker detection (Ghersevich et al., 2015). This technical heterogeneity complicates comparisons between studies and limits the translation of findings into clinically applicable markers.

In summary, while FF-EVs constitute a promising source of non-invasive indicators of reproductive competence, current evidence is largely correlative and exploratory. Future studies should prioritize standardized methodologies, longitudinal designs, and functional validation in physiologically relevant models to establish whether specific FF-EVs components can serve as reliable and clinically meaningful biomarkers.

### 2.2 Effect of external follicular factors on EVs secretion and contents

EVs secretion is a regulated process influenced by cellular state and external environmental stimuli, with selective cargo loading mechanisms (van Niel et al., 2018). In the ovarian follicle, both the molecular composition and abundance of FF-EVs vary according to follicular stage and conditions, supporting the concept of a dynamic vesicle-mediated communication system (Navakanitworakul et al., 2016). For example, small growing follicles show higher EVs concentration and distinct miRNA (Gebremedhn et al., 2020), and protein profiles compared to larger preovulatory follicles. Functionally, FF-EVs isolated from smaller follicles have been reported to more effectively promote CCs expansion (Hung et al., 2015), although the mechanisms underlying these differences such as selective uptake or inhibitory cargo remain unresolved and have not been validated *in vivo*.

Under oxidative stress, granulosa cell-derived EVs are enriched in NRF2 and antioxidant enzymes, both reflecting and counteracting cellular damage (Gebremedhn et al., 2020), while heat stress induces EVs-mediated secretion of heat shock proteins, which confer resistance to recipient cells and reduce apoptosis and DNA damage, as a “bystander effect” (Bewicke-Copley et al., 2017) (Figure 3). Although supplementation with FF or FF-EVs can partially mitigate heat stress effects on oocyte developmental outcomes *in vitro*, these findings should be interpreted cautiously, as EVs concentrations and exposure conditions may not reflect physiological scenarios. Similarly, metabolic stress alters FF-EVs cargo composition: negative energy balance in postpartum cows, is associated with dowregulation of specific miRNAs such as bta-miR-132, bta-miR-34b/c, and bta-miR-449a (Hailay et al., 2019), while body energy reserves correlate with distinct EVs miRNA signatures (Bastos et al., 2023). However, these changes are primarily descriptive, and direct causal links to oocyte competence are still lacking.

Beyond nucleic acids, FF-EVs also carry lipid species associated with oocyte competence, suggesting potential biomarkers for developmental capacity (da Silveira et al. (2021). Across studies, a key limitation is the high sensitivity of EVs profiles to methodological variables, including isolation techniques and experimental conditions, which complicates interpretation of stress-induced changes and limits cross-study comparability.

Overall, FF-derived EVs appear to serve as a responsive and potentially adaptive communication system, modulating their cargo to reflect and respond to environmental stressors. Whether these changes are beneficial or detrimental likely depends on the duration, intensity, and timing of the stress. For instance, under moderate stress, EVs-mediated responses may protect oocytes and somatic cells (adaptive), while under chronic or severe stress, the changes in EVs cargo may signal damage and compromise developmental outcomes (maladaptive or neutral). To provide clarity and synthesis, we include a summary table (Supplementary Table S3) that contrasts EVs content and observed effects under different stressors and follicular stages.

Collectively, these findings support the concept that FF-derived EVs act as dynamic and context-dependent regulators of follicular communication, modulating oocyte competence and metabolic responses according to physiological and environmental conditions. This adaptive capacity highlights the broader role of EVs-mediated signaling within the reproductive tract. Future work should focus on standardized methodologies and mechanistic studies in physiologically relevant models to determine whether stress-induced changes in EVs cargo actively regulate follicular function or primarily serve as biomarkers of cellular state.

## 3 The role of EVs in sperm maturation

Spermatogenesis begins in the seminiferous tubules of the testes, where primordial germ cells differentiate into spermatozoa that are morphologically formed but functionally immature upon release into the lumen (James et al., 2020). Acquisition of fertilizing capacity takes place during epididymal transit, a process involving coordinated structural and molecular remodeling, including changes in chromatin condensation, remodeling of membrane proteins, and changes in phospholipid and cholesterol content (Sullivan and Saez, 2013; Gervasi and Visconti, 2017). Increasing evidence implicates EVs as contributors to these processes; however, their precise functional roles and relative importance compared to other mechanisms remain incompletely defined.

Epididymosomes, EVs secreted by the epididymal epithelium, have been proposed as key mediators of sperm maturation through the transfer of proteins, lipids, and small RNAs including miRNAs and tRNA-derived fragments directly to spermatozoa (Sullivan, 2015) (Figure 2). Recent studies in cattle demonstrate that spermatozoa can internalize EVs from seminal plasma, which carry fertility-associated proteins and contribute to the modulation of sperm functional competence (Pal et al., 2025). Their content and functions are region-specific:

- In the caput epididymis, epididymosomes are enriched in proteins like P25b, MIF, and miRNAs such as mmu-miR-145, mmu-miR-143, and mmu-miR-214, which are involved in early remodeling of the sperm membrane and chromatin (Belleannée et al., 2013).
- In the cauda epididymis, epididymosomes contain different regulatory molecules (e.g., mmu-miR-654, mmu-miR-1224), which may be involved in preparing sperm for storage and subsequent ejaculation (Belleannée et al., 2013; Sullivan and Saez, 2013).

In contrast, prostasomes are EVs secreted by the prostate gland into the seminal plasma and interact with sperm primarily after ejaculation, not during epididymal transit. They are generally larger (40–500 nm) and enriched in cholesterol, calcium, and membrane fusion proteins (Ronquist and Brody, 1985; Andrews et al., 2015). Prostasomes primarily bind to the sperm midpiece and plasma membrane, enhancing motility and hyperactivation; these effects are often attributed to modulation of calcium signaling and delivery of antioxidant components (Andrews et al., 2015; Xu et al., 2024). However, as with epididymosomes, much of the evidence derives from *in vitro* supplementation experiments, which may not accurately reflect physiological concentrations or exposure dynamics. Consequently, the extent to which prostasomes contribute to sperm function under *in vivo* conditions remains uncertain.

These functional distinctions between prostasomes and epididymosomes highlight stage-specific interactions of EVs with sperm where epididymosomes are essential during maturation, while prostasomes modulate sperm function after ejaculation. Current evidence supports a role for EVs-mediated protein transfer in sperm remodeling, but its contribution as a primary mechanism for establishing sperm competency, promoting embryogenesis, and facilitating epigenetic inheritance remains insufficiently demonstrated (Supplementary Table S1). In contrast, the role of EVs associated with small RNAs (e.g., miRNAs, tRNA fragments) is even less defined, relying largely on indirect or correlative data with minimal functional validation. Addressing these gaps will require quantitative *in vivo* analyses of EVs - sperm interactions, and functional studies linking specific EVs cargo to reproductive outcomes.

### 3.1 The effect of EVs on fertilization

Fertilization is a tightly regulated process that occurs in the oviduct, anatomically divided into the infundibulum, ampulla and isthmus. Although fertilization typically occurs in the ampulla, the isthmus acts as a sperm reservoir where critical molecular changes take place, preparing sperm for fertilization and early embryonic development. Emerging evidence indicates that EVs secreted by the oviductal epithelium, oocytes, and CCs contribute to this process; however, these findings are largely based on *in vitro* models and require further *in vivo* validation.

#### 3.1.1 Oviductal EVs (OF-EVs) and sperm capacitation

Before fertilization, sperm must undergo capacitation, acrosome reaction, penetration of the zona pellucida and membrane fusion. Emerging evidence suggests that oviductal fluid-derived EVs (OF-EVs) contribute to these processes, as bovine studies report increased tyrosine protein phosphorylation and enhanced intracellular Ca^2+^ regulation facilitating capacitation and acrosome reaction (Franchi et al., 2020). However, these findings are predominantly derived from *in vitro* systems, and their physiological relevance and magnitude *in vivo* remain uncertain.

On the other hand, membrane fusion between sperm and oocyte is a highly specific event. Mouse studies have shown that EVs containing CD9 and CD81, released from the oocyte membrane, are critical for successful sperm-oocyte fusion (Miyado et al., 2008; Tanigawa et al., 2008; Ohnami et al., 2012). Functional evidence further supports a role for CD9, as CD9 deficient oocytes fail to fuse unless complemented by EVs from wild-type oocytes, suggesting its transferability (Miyado et al., 2008), while CD8, 1 primarily localized to the zona pellucida, may also facilitate acrosomal exocytosis (Tanigawa et al., 2008). Following fertilization, oocytes release Juno-containing EVs into the perivitelline space, which act as decoys by binding excess sperm and preventing polyspermy (Bianchi and Wright, 2014b; Bianchi et al., 2014a).

Apart from peptides, proteins, enzymes, hormones, cytokines, lipids, sugars, and ions, EVs interacting with spermatozoa are also part of the seminal plasma (Machtinger et al., 2016). Proteomic studies in several species identified additional EVs components (e.g., ATP1A4, PGK2, TEKT3), involved in motility, zona pellucida recognition, and metabolic regulation (Alcântara-Neto et al., 2020b; Harris et al., 2020). Similarly, oviductal EVs associated proteins such as OVGP1, SPAM1, and PMCA4 have been linked to improve sperm viability, motility, and cumulus matrix penetration (Griffiths et al., 2008a; Almiñana et al., 2017; 2018). Among these, PMCA4 has been proposed to regulate intracellular Ca^2+^ homeostasis and support hyperactivation and prevent premature capacitation, in part by inhibiting Ca^2+^-dependent nitric oxide production (Al-Dossary et al., 2013; Andrews et al., 2015); however, most supporting evidence relies on indirect functional assays rather than direct demonstration of EVs-mediated transfer and activity *in vivo*. Likewise, the roles of OVGP1 and SPAM1 support fertilization by increasing sperm viability, motility, and dispersal through CCs (Griffiths et al., 2008a; Almiñana et al., 2017), but their specific contribution via EVs, as opposed to soluble forms, remains difficult to disentangle. Overall, although EVs are increasingly proposed as modulators of sperm capacitation and fertilization, current evidence is largely associative or based on *in vitro* supplementation models. Future studies should focus on standardized approaches and direct functional validation to clarify the extent to which EVs-mediated cargo transfer drives sperm function under physiological conditions.

## 4 EVs and their implication in early embryo development

Successful early embryo development, implantation and maintenance of a pregnancy depend on effective embryo-maternal communication. EVs have emerged as potential mediators of this crosstalk, facilitating the transfer of bioactive molecules such as mRNAs, miRNAs, and proteins (Figure 1; Supplementary Table S1). These molecules help enhance embryo development and may increase the efficacy of assisted reproductive technologies (ARTs). EVs are secreted by both maternal tissues and embryos and are exchanged during gamete transit and embryo development through the oviduct and uterus. This exchange is associated with molecular communication processes in which EV-associated miRNAs modulate pathways related to embryo development, lipid metabolism, and immune regulation (Mazzarella et al., 2024).

Importantly, EV-mediated signaling is highly sensitive to experimental conditions, and *in vitro* culture systems can alter both EVs composition and embryo-maternal communication dynamics (Aguilera et al., 2024). This variability complicates interpretation of functional outcomes and highlights the need to clearly distinguish between *in vitro* observations and *in vivo* biology, a distinction that is often overlooked. The following sections therefore critically separate these contexts to elucidate EVs roles in embryo-maternal communication during early development.

### 4.1 EVs derived from female reproductive tract: *in vitro* and *in vivo* evidence

EVs were first reported in UF from mice in 2008 (Griffiths et al., 2008a) and in equine FF in 2012 (da Silveira et al., 2012). Since then, numerous studies have identified EVs in OF, UF and conditioned culture media from oviductal and endometrial epithelial cells across multiple species, including humans, cattle, pigs, horses, and small domestic animals (Ng et al., 2013; Krawczynski et al., 2015; Lange-Consiglio et al., 2017; Lopera-Vasquez et al., 2017; Almiñana et al., 2018; 2021; Bathala et al., 2018; Kusama et al., 2018; Qiao et al., 2018; Ferraz et al., 2019; Qu et al., 2019).

*In vivo* studies show that EVs cargo varies with estrous cycle stage and pregnancy status, affecting miRNA (Almiñana et al., 2018), lipids (Banliat et al., 2020; O’Neil et al., 2020) and metabolites (Gatien et al., 2019) cargoes. For instance, Mazzarella et al. (2021) found distinct miRNA profiles in EVs from pregnant and non-pregnant bovine oviducts, suggesting an embryo-induced modulation of EVs content. Similarly, Hamdi et al. (2021) also observed cycle-dependent miRNA variations in both OF and UF-EVs. In line with these observations, recent evidence demonstrates that EV-associated miRNAs from the oviduct and uterus can modulate signaling pathways related to lipid metabolism and early embryo development in cattle (Mazzarella et al., 2024).

*In vitro* studies provide more direct, but still limited, functional insights. EVs from OF or BOECs are internalized by multiple cell types, including sperm, oocytes, CCs, preimplantation embryos, and endometrial epithelial cells (Al-Dossary et al., 2013; Alcântara-Neto et al., 2020a; Franchi et al., 2020; Lee et al., 2012; Griffiths et al., 2008b; Ruiz-González et al., 2015; Burns et al., 2016; Greening et al., 2016; Evans et al., 2019). Additionally, embryos also secrete EVs that modulate maternal epithelial cell activity, supporting a model of bidirectional communication critical for early development and pregnancy establishment. Nevertheless, these studies often rely on EVs supplementation or co-culture systems, where vesicle concentrations, exposure times, and isolation methods vary widely, potentially confounding functional interpretations.

Recent proteomic analyses suggest that the presence of the embryo can alter the molecular composition of oviductal EVs, supporting the concept of embryo-driven modulation of the maternal environment (Mazzarella et al., 2025b). Yet, the mechanisms underlying this interaction, including how EVs cargo is selectively packaged, transferred, and functionally integrated into recipient cells, remain largely unresolved.

Overall, while EVs are consistently implicated in embryo–maternal communication, current evidence is dominated by descriptive profiling and *in vitro* experimentation. Methodological variability in EVs isolation, characterization, and experimental design further limits reproducibility and cross-study comparisons. Addressing these limitations through standardized approaches and *in vivo* functional validation will be essential to determine whether EVs actively regulate early embryo development or primarily serve as indicators of physiological state.

### 4.2 Embryo–maternal interactions through oviductal EVs

The oviduct provides the environment for early embryo development, including the first cleavages and genome activation. A substantial body of evidence suggests that oviductal EVs influence embryo development; however, most of this evidence derives from *in vitro* supplementation studies. For instance, EVs from oviductal epithelial cells (OECs) or OF have been associated with enhanced blastocyst quality, including increased blastocyst cell numbers, cryotolerance, mitochondrial activity, and reduced apoptosis, ROS levels and modulation of quality-related genes (Lopera-Vásquez et al., 2016; Leal et al., 2022). Despite these consistent observations, not all studies report beneficial effects, as some show no improvement in embryo quality but only changes in transcriptomic or lipid profiles (Banliat et al., 2020; Bauersachs et al., 2020). This variability likely reflects differences in EVs isolation methods, culture conditions, and experimental design, complicating direct comparisons across studies. Mechanistically, the functional contribution of EVs cargo remains insufficiently resolved. More recently, Xue et al. (2024) reported improved embryo development, hatching, and metabolic profiles with BOEC-derived EVs, highlighting their influence on key gene pathways. Besides, many genes related to embryo development and quality, antioxidants and energy metabolism were modulated (Sidrat et al., 2020; Leal et al., 2022).

Although *in vivo* data are scarce, embryonic presence appears to modulate maternal EVs output. For example, Mazzarella et al. (2021), reported changes in miRNA content in OF and BOECs EVs from cows at Day 5 of pregnancy, suggesting that embryos may modulate maternal EVs production very early. This concept was further strengthened by Mazzarella et al. (2025a), who identified alterations in the protein cargo of oviductal EVs in both *in vivo* and *ex vivo* models. These EVs contained proteins associated with embryo interaction and early development (CUL1, DHX15, and PRMT1), DNA repair (UBA2 and RAD50), cell differentiation (HEBP1 and RPS21), metabolic regulation (MDH1, IDH1, and TPI1), and embryogenesis-related processes such as adhesion and cytoskeletal remodeling (TAGLN2 and ITGB1). Importantly, comparison between *in vivo* and explant-based *in vitro* models showed that both systems captured embryo-induced changes in EVs cargo, supporting their relevance of the *ex vivo* model for studying embryo–maternal communication in the oviduct.

Furthermore, embryo-derived EVs have been shown to modulate BOEC gene expression, particularly pathways related to interferon tau (IFNτ) signaling, a crucial pregnancy recognition pathway (Dissanayake et al., 2021). However, the extent to which this bidirectional EVs exchange drives functional outcomes *in vivo*, as opposed to reflecting parallel signaling processes, is still unclear. Overall, while oviductal EVs are increasingly implicated in early embryo-maternal communication, current evidence is heterogeneous and largely based on *in vitro* systems, underscoring the need for standardized methodologies and *in vivo* functional validation.

### 4.3 Embryo–maternal interactions through uterine EVs

Following oviductal transit, embryos encounter uterine EVs during the peri-implantation period, a critical window for establishing pregnancy. After the first days in the oviduct, the embryos encounter uterine EVs during the peri-implantation window (e.g., days 16–22 in bovine (Kusama et al., 2018; Malo Estepa et al., 2020; Nakamura et al., 2020; Nakamura et al., 2016), days 10–18 in pigs (Krawczynski et al., 2015; Bidarimath et al., 2017; Hua et al., 2021) and days 10–17 in ovine (Burns et al., 2014; Ruiz-González et al., 2015; Nakamura et al., 2020; O’Neil et al., 2020; Nakamura et al., 2016). These EVs have been associated with processes such as blastocyst development, proliferation, hatching, and anti-apoptotic signaling, however, much of the supporting evidence comes from *in vitro* or *ex vivo* models (Supplementary Table S1).

*In vitro* studies show that uterine EVs, particularly those collected during early CL stages (Days 5–10 in cattle), can enhance blastocyst development, proliferation, hatching, anti-apoptotic signaling (Lv et al., 2018; Qiao et al., 2018) and modulate gene expression in key pathways related to IFN-τ signaling and acrogranin (associated with invasion), with reduced BAX and elevated Bcl-2/Oct4 levels (Lv et al., 2018).

Recent *in vivo* and *ex vivo* evidence indicates that embryos can actively modulate uterine EVs composition. Mazzarella et al. (2025a) showed that the presence of a single blastocyst at day 7 of pregnancy is associated with alterations in uterine EVs protein cargo, observed in both *in vivo* and *in vitro* systems. *In vivo*, EVs from pregnant heifers were enriched in proteins linked to embryonic development, immune and inflammatory regulation, and endometrial receptivity. These include extracellular matrix components (LAMB1, COL1A2, and LAMC1), associated with tissue remodeling and embryo-endometrium interaction, as well as CRB2, involved in epithelial polarity and lineage specification. Additional proteins such as UFL1, HSPA2, TTLL12, and PSMB2 suggest potential roles in immune modulation and cellular homeostasis; however, these associations are based on proteomic profiling and lack direct functional validation.

In parallel, *in vitro* models using endometrial explants co-culture models with embryos revealed embryo- associated changes in EVs protein cargo, but with partially divergent protein profiles, including factors related tocell differentiation and polarity (NELFB, THOC5, CXADR, and CLDN7), interferon-mediated signaling (SCRN1, TUBA1A, MLEC, EIF2AK2, ERAP2, GNB4, PPA1, and SCP2), and endometrial receptivity (LCN2 and PTGES2). Although the protein profiles differed between *in vivo* and *in vitro* conditions, a substantial proportion of proteins identified in both systems were similarly abundant, supporting the physiological relevance of the explant model to study early embryo-maternal communication.

Collectively, these findings indicate that uterine EVs actively participate in embryo-maternal communication by delivering bioactive molecules that regulate key processes including cell polarity, adhesion, immune modulation, and tissue remodeling. (Figure 1; Supplementary Table S1). While some overlap between *in vivo* and *in vitro* datasets support the utility of explant models, discrepancies highlight the sensitivity of EVs composition to experimental conditions. Thus, although these studies support embryo-dependent modulation of uterine EVs, it remains unclear whether these changes actively regulate implantation or primarily reflect underlying physiological processes.

### 4.4 EVs derived from embryo and their cargoes on early embryo development

As mentioned above, embryo–maternal communication is a bidirectional process; however, the specific contribution of embryo-derived EVs to maternal modulation remains incompletely defined. After fertilization, the embryo stays in the oviduct until Day 3.5–4, interacting with OF and epithelial cells (Figure 1). This is a crucial period of embryo development because it includes embryonic genome activation, which occurs at the 8- to 16-cell stage in the cattle (Gad et al., 2012), and is critical for future cell differentiation, embryo implantation, and fetal development. Although this period is often proposed as highly responsive to EVs-mediated signaling, direct *in vivo* evidence remains limited.

*In vivo*, Maillo et al. (2015), found minimal transcriptomic changes in the oviduct due to the presence of a single eight-cell embryo, but they observed shifts in immune related gene expression, suggesting that the embryo may actively modulate the immune system in the maternal tract avoiding its own rejection by decreasing inflammation and antigen presentation. Years later, Rodríguez-Alonso et al. (2020) demonstrated proteomic and metabolic changes in OF associated with presence of an 8-cell embryo, reflecting its immediate environmental needs. More recent studies further show that embryo presence is associated with alterations in oviductal EVs cargo, including proteins associated with genome activation, DNA repair, cell differentiation, migration, and immune tolerance, highlighting a dynamic and embryo-driven modulation of the maternal environment (Mazzarella et al., 2025a). However, these findings are primarily descriptive and do not establish whether such changes are functionally required for embryo development or represent secondary responses.

Nevertheless, changes in oviductal transcriptome and metabolites profiles across the estrous cycle, along with alterations in the expression of selected genes triggered by embryos during the preimplantation period, reinforce the view that the embryo actively influences oviductal cells, that adapts its environment according to its changing needs (Lopera-Vasquez et al., 2017; Rodríguez-Alonso et al., 2019; Almiñana and Bauersachs, 2020; Bauersachs et al., 2020).

*In vitro* studies provide additional, but still limited, mechanistic insights. Hamdi et al. (2019) observed that early bovine embryos altered BOEC gene expression in a stage-specific manner. In addition, the miRNA cargo of oviductal EVs has been shown to vary depending on embryo presence and quality, supporting a role for EVs in embryo recognition and the regulation of early embryo–maternal communication (Hamdi et al., 2024). Wydooghe et al. (2017) described embryotropins as bioactive molecules, including proteins, lipids, and miRNAs secreted by mammalian embryos. Similarly, embryo-derived EVs have been shown to modulate immune-related genes (e.g., ISG15, MX1, OAS1Y) and interferon tau (IFNτ) signaling in recipient cells (Dissanayake et al., 2021), supporting a role in maternal recognition processes.

Specific EVs-associated miRNAs have been proposed as regulators of embryo development. Mazzarella et al. (2021) found 8 miRNAs (bta-miR-126-5p, bta-miR-129, bta-miR-140, bta-miR-188, bta-miR-219, bta-miR-345-3p, bta-miR-4523, and bta-miR760-3p) upregulated in oviductal flushing from pregnant cows; while, Pavani et al. (2022) showed that bta-miR-378a-3p, which was upregulated in EVs secreted by blastocysts, promotes blastocyst hatching and impacts gene expression related to embryo development and implantation such as: *RAP1GAP*, *ARFGEF2*, *SLC7A6*, *CENPA*, *SP1*, *LDLR*, *PYCR1*, *MYD88*, *TPP*1, and *NCOA3*, identifying miR-378-3p as a potential biomarker of embryo quality and implantation success, and bta-miR-148b, has been shown to enhance embryo quality and modulate the TGF-β signaling pathway by regulating key genes involved in cell proliferation and pluripotency, including SMADs, TGFBR2, POU5F1, and NANOG (Cañón-Beltrán et al., 2024). Despite these examples, functional validation is limited to a small number of candidates, and most proposed roles of EVs associated miRNAs are inferred from expression data or indirect assays.

Importantly, EV-mediated embryo–maternal communication appears highly sensitive to *in vitro* conditions, which can alter EVs cargo, IFNτ expression, and developmental outcomes (Aguilera et al., 2024). This highlights a key limitation of the field, as methodological variability in EVs isolation, characterization, and experimental design may significantly influence reported effects. This highlights a key limitation of the field, as methodological variability in EVs isolation, characterization, and experimental design may significantly influence reported effects. Overall, while embryo-derived EVs are increasingly implicated in shaping the maternal environment, current evidence remains largely correlative and model-dependent. Future studies should prioritize *in vivo* functional approaches to determine whether specific EVs cargoes play causal roles in early embryo development or primarily reflect embryo quality and physiological state.

## 5 Mechanisms of EV-mediated signaling in the oviduct and uterus

Despite numerous studies describing beneficial effects of EVs on early embryo development and maternal responses, the underlying molecular mechanisms remain poorly defined and are often inferred rather than directly demonstrated. Current evidence highlights miRNAs as key modulators via the RNA-induced silencing complex (RISC), binding to complementary sequences within the 3’ untranslated regions (UTRs) of target mRNAs, regulates target mRNAs by degradation or translational inhibition, thereby influencing pathways related to immune modulation, cellular stress response, apoptosis, and metabolism (Bridi et al., 2020; Melo-Baez et al., 2020; Tan et al., 2020; Maraghechi et al., 2023; Shang et al., 2023; Mazzarella et al., 2024; Zheng et al., 2025). However, most studies rely on predicted targets or indirect transcriptomic correlations, with limited evidence confirming functional delivery and activity of these miRNAs in recipient cells *in vivo*.

In the bovine oviduct, embryo-derived EVs have been shown to influence BOECs, inducing the modulation of IFN-τ signaling pathway, including ISG15, MX1, and OAS1Y promoting immune tolerance (Dissanayake et al., 2021). Additionally, Mazzarella et al. (2021) demonstrated that specific EVs associated miRNAs (bta-miR-126-5p, bta-miR-129, bta-miR-140) have been reported to vary under pregnant conditions and are predicted to target genes involved in immune and proliferative genes. Nevertheless, these associations are largely based on expression analyses and lack direct mechanistic validation. A limited number of studies provide functional evidence; for example, Pavani et al. (2022) demonstrated that bta-miR-378a-3p (secreted in EVs from high quality bovine blastocysts), enhances hatching and modulates genes involved in development and implantation, including *RAP1GAP, ARFGEF2, SLC7A6, CENPA, SP1*, and *MYD88*, indicating a broad regulatory role of EVs-contained miRNAs in shaping embryonic competence. Even in these cases, the specific contribution of EVs-mediated delivery, as opposed to other signaling mechanisms, remains difficult to isolate.

In addition, experimental evidence from other mammalian systems has shown that trophectoderm-derived EVs can alter the proteomic profile of endometrial epithelial cells, supporting the idea that EVs cargo actively modulates maternal cellular responses during embryo implantation (Poh et al., 2021).

In the uterus, EVs have been associated with modulation of embryonic and maternal gene expression, including upregulating IFN-τ, acrogranin, and Oct4 levels, while reducing expression of stress-related markers (*BAX, HSP70*) (Lv et al., 2018; Qiao et al., 2018). However, these findings are predominantly derived from *in vitro* supplementation studies, where EVs concentration, and exposure conditions vary considerably, potentially influencing the observed effects. More broadly, EVs have been implicated in the establishment a tolerogenic maternal environment, reinforcing their role in coordinating embryo survival and implantation-associated processes (Godakumara et al., 2021), but the relative contribution of EVs-mediated signaling compared to other endocrine and paracrine pathways remains unclear.

**FIGURE 4:**
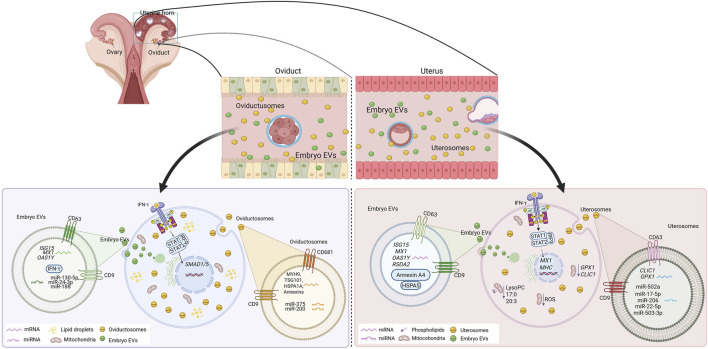
Extracellular vesicle (EVs) mediated embryo–maternal communication during early development. EVs mediate bidirectional signaling between the embryo and maternal tract by transferring bioactive molecules that regulate embryo development, immune tolerance, and implantation. EV-associated miRNAs modulate key gene networks, enhancing developmental competence and supporting pregnancy establishment. Elements are schematic and not drawn to scale. Figure created with BioRender.com.

Collectively, current evidence supports a model in which EVs act as carriers of bioactive molecules, including miRNAs, that contribute to embryo-maternal communication; by modulating gene regulatory networks involved in maternal environment adaptation, and embryo development. However, this model is largely based on correlative data and simplified *in vitro* systems, limiting its physiological interpretation. From the oviduct to the uterus, EVs are proposed to mediate the transfer of bioactive molecules that regulate embryo developmental competence, maternal cellular responses, and endometrial receptivity for implantation (Figure 4). Despite valuable mechanistic insights from *in vitro* studies, their relevance under *in vivo* conditions remains uncertain. Future research should focus on integrating multi-omics approaches with physiologically relevant *in vivo* models to elucidate the specificity and functional impact of EVs-mediated signaling. Such advances will be critical to determine whether EVs can be reliably exploited to improve assisted reproductive technologies and reproductive efficiency in cattle.

## 6 EVs and their potential roles during conceptus elongation and maternal recognition of pregnancy

Conceptus elongation in cattle starts around days 12–19 post-estrus and involves rapid trophectoderm expansion, remodeling of extraembryonic membranes, and a significant increase in conceptus size (Lonergan et al., 2016). This process is regulated by progesterone (P4), gene expression (Spencer et al., 2016), and UF which promotes trophectoderm cell growth and migration (Brooks et al., 2014) and provides growth factors, carbohydrates, lipids, amino acids, and growth factors (Simintiras et al., 2019). Although UF is clearly required for elongation, the specific contribution of its EVs cargo in supporting development remains incompletely defined and is often inferred from indirect evidence.

As we mentioned in the previous section, EVs in the UF increase during early pregnancy and can be internalized by both the trophectoderm and endometrial epithelial cells (EECs), delivering RNAs, and proteins that promote elongation through cell proliferation and migration (Burns et al., 2016; Kusama et al., 2018). For example, UF EVs isolated from day 14 pregnant sheep contain a complex cargo of 195 proteins, 81 conserved mature miRNAs, and 512 mRNAs, including ribosomal components (Burns et al. (2016). While these findings support a role for EVs in intercellular communication, they are largely descriptive and do not directly demonstrate functional effects on elongation *in vivo*.The elongation period coincides with a critical window of embryonic loss, often associated with insufficient IFNτ signaling or impaired embryo-uterine communication (Neupane et al., 2017). IFNτ secreted by the elongating conceptus, prevents luteolysis by inhibiting prostaglandin F2 alpha (PGF2α) release, thereby maintaining P4 production (Brooks and Spencer, 2015). EVs have been proposed as additional mediators of this process; however, their relative contribution compared to soluble factors such as IFNτ remains unclear.

Several studies report that UF-EVs from pregnant animals contain IFNτ and can induce interferon-stimulated genes (ISGs) like ISG15, MX1/2, and STAT1/2 in EECs, expression of apoptosis-related genes such as *TNFA*, *BAX*, *TP53*, and *CASP3* and other immuno-modulatory proteins, as well as CAPG and AKR1B1. Notably, these effects have been primarily demonstrated *in vitro*, where EVs exposure leads to changes in gene expression, including apoptosis-related pathways, but their physiological relevance and dosage dependency *in vivo* are not well established.

Recent findings indicate that EVs cargo varies depending on embryo origin. For instance, EVs from *in vitro*-produced embryos induce broader transcriptomic changes in endometrial cells compared to those derived *in vivo* (Aguilera et al., 2024). This highlights a critical methodological limitation, as *in vitro* conditions may alter EVs composition and exaggerate their apparent functional effects. Additionally, specific EVs-associated miRNAs such as bta-miR-499 and bta-miR-98 modulate immune responses, promoting a tolerant uterine environment during elongation (Nakamura et al., 2019; Zhao et al., 2019). Overall, EVs are increasingly proposed as modulators of conceptus elongation and maternal recognition of pregnancy; however, current evidence is predominantly correlative and heavily dependent on *in vitro* models. Greater mechanistic insight will require quantitative *in vivo* studies, and direct functional validation to determine whether EVs-mediated signaling plays a causal role or primarily reflects ongoing embryo-uterine interactions.

## 7 EVs and their potential roles during implantation and gestation

Following conceptus elongation and maternal recognition of pregnancy, implantation represents the next pivotal step, involving apposition, adhesion, and attachment of the trophectoderm to the endometrial luminal epithelium (Campanile et al., 2021; Green et al., 2021). Successful implantation requires endometrial receptivity and a dynamic, bidirectional communication between the conceptus and uterus, processes that have been increasingly associated with EVs, but direct *in vivo* functional validation remains limited (O’Neil et al., 2020; Hart et al., 2022).

EVs derived from the endometrium have been proposed to deliver bioactive molecules such as miRNAs and proteins, to the conceptus and adjacent endometrial cells, potentially influencing apoptosis, immune regulation, and cellular adhesion. For instance, Kusama et al. (2018) identified 172 differentially expressed proteins in UF-EVs from days 17, 20, and 22 of pregnancy in cattle, with pre-implantation EVs associated with increased expression of adhesion-related markers like VCAM1 in endometrial epithelial cells (EECs). While these findings suggest a role in modulating endometrial receptivity, they are largely based on proteomic profiling and cell culture assays and therefore should be interpreted as indicative rather than definitive evidence of functional relevance *in vivo*. Similarly in humans, endometrial EVs have been shown to enhance trophoblast adhesion through FAK signaling and fibronectin production (Greening et al., 2016), but these observations may not fully recapitulate the complexity of the implantation environment (Giacomini et al., 2017).

EVs-associated miRNAs have also been implicated in regulating implantation-related processes, including integrin signaling, extracellular matrix remodeling, and immune response (Liang et al., 2017). Human endometrial EVs carry has-miR-200c, has-miR-17, and has-miR-106a, which contribute to adhesion and migration (Ng et al., 2013), while in cattle, bta-miR-26b has been associated with downregulation of immune-related genes such as *PSMC6*, *CD40*, and *IER3* in EECs (Nakamura et al., 2021). Nevertheless, these studies primarily demonstrate molecular associations or effects in controlled experimental settings, and causal links between specific EVs cargo and implantation success remain to be firmly established. In addition, UF-EVs from day 20 pregnant cows have been reported to modulated neutrophils function, influencing maternal immunity and implantation (Nakamura et al., 2021), but the underlying mechanisms and their physiological significance *in vivo* are still poorly understood and warrant further investigation.

Beyond implantation, EVs continue to facilitate maternal–fetal communication during gestation, supporting immune tolerance and placental development. Circulating EVs have also been explored as potential biomarkers of pregnancy status and viability (Pohler et al., 2017; Markkandan et al., 2018). For instance, differential abundance of specific miRNAs has been reported in serum EVs from cows with embryonic mortality on day 17, and similar trends were observed in SCNT pregnancies (De Bem et al., 2017; Pohler et al., 2017), as well as between pregnant and cyclic cows at day 30 (Markkandan et al., 2018). Stage specific EVs-associated miRNA profiles have also been described throughout gestation, for example, bta-miR-499, bta-miR-16a and bta-miR-20a were present at day 60; bta-miR-493, bta-miR-127, and bta-miR-143 at day 150; and bta-miR-122, bta-miR-182, bta-miR-183, bta-miR-200b, and bta-miR-200c at day 240 of gestation (Zhao et al., 2019). Importantly, the EVs field is particularly sensitive to methodological variability that can substantially affect EVs yield, purity, and molecular composition, thereby influencing downstream functional interpretations. Many of the studies discussed here employ distinct and sometimes non-standardized methodologies, which may contribute to inconsistencies across findings and limit reproducibility regarding their roles in implantation and gestation.

Collectively, these observations suggest that EVs are associated with key processes during implantation and gestation, such as adhesion, immune modulation, and maternal-fetal communication. and reflecting pregnancy status through their circulating cargo. However, the field currently relies heavily on associative evidence, and there is a clear need for more rigorous *in vivo* and mechanistic studies to delineate causal roles and underlying pathways. Addressing these gaps will be critical to move from descriptive observations toward a more comprehensive and mechanistic understanding of EVs function in reproductive biology.

## 8 Future implications of EVs and their role in enhancing ARTs

EVs carry diverse molecular cargoes including miRNAs, mRNAs, proteins, and metabolites which have been implicated in the regulation of gene expression and cellular responses. In the context of bovine *in vitro* fertilization (IVF), interest in EVs has increased due to their proposed dual application as non-invasive biomarkers and as modulators of embryo development and implantation; however, their performance as reliable biomarkers has not yet been established, and much of the current evidence remains exploratory and context-dependent.

EVs secreted by embryos have been proposed to reflect their developmental competence. For example, Dissanayake et al. (2020) found that EVs collected at different developmental stages (days 2 and 8) exhibit stage-associated differences with potential diagnostic relevance, while Mellisho et al. (2019) observed higher EVs concentrations in non-viable bovine blastocysts compared to viable ones. Additionally, Melo-Baez et al. (2020) identified 95 bta-miRNAs in culture media from embryos arrested at the 8–16-cell stage. Specific miRNAs, such as bta-miR-1, bta-miR-10b, and bta-miR-184, have been associated with early developmental arrest (Lin et al., 2019; Mellisho et al., 2019; Zhang et al., 2019), and in humans, hsa-miR26b-5p and hsa-miR-21-5p have been correlated with successful pregnancies (Fang et al., 2021). However, these studies primarily report associations and do not provide quantitative evaluation of predictive performance (e.g., sensitivity, specificity, or accuracy) for outcomes such as embryo arrest or pregnancy success. Thus, the actual predictive value of these miRNAs as biomarkers remains unclear.

Despite these associations, most studies are based on correlative analyses of spent culture media, and causal relationships between EVs cargo and embryo viability have not been conclusively demonstrated. Factors such as embryo culture conditions, EVs isolation techniques, sample volume, and RNA profiling platforms can significantly influence EVs yield and cargo composition, thereby limiting reproducibility and hindering robust evaluation of biomarker performance. These challenges currently limit the translation of EVs-based diagnostic approaches into clinical IVF settings.

In this context, engineered or “artificial EVs” are emerging as a promising strategy to improve standardization and reproducibility with the development of EVs-mimetic systems with defined cargo and controlled bioactivity, including bioengineered exosomes and nanovesicles (El Andaloussi et al., 2013; Yáñez-Mó et al., 2015; Vader et al., 2016). In bovine ARTs, artificial EVs may serve as controlled systems to study embryo–maternal communication and to support biomarker validation by reducing biological variability. However, their application in reproductive systems remains at an early stage, and key aspects such as safety, stability, biodistribution, and functional equivalence to natural EVs require further investigation. Indeed, evidence from other fields indicates that engineered EVs may differ from native vesicles in uptake efficiency and biological effects (Wiklander et al., 2019). Therefore, their implementation in ARTs will require rigorous validation and standardized production protocols to ensure biological relevance.

### 8.1 EVs as therapeutic delivery vehicles in IVF culture systems

EVs have also been proposed as delivery systems to enhance embryo development and optimize *in vitro* culture conditions. Pavani et al. (2018) reported that EVs can penetrate the ZP and improve bovine embryo quality by reduction in apoptotic cells, while Leal et al. (2022) showed that sequential supplementation with OF and UF-EVs modulates the expression of genes related to mitochondrial function and lipid metabolism. Similarly, Lopera-Vásquez et al. (2016) showed that *in vitro* EVs supplementation upregulated *GJA1* and *GAPDH*, genes linked to embryo quality and cryotolerance.

While these findings suggest a functional role for EVs in improving embryo competence, most evidence derives from *in vitro* supplementation, often using heterogeneous EVs populations with variable purity and origin. As a result, it remains unclear which specific EVs subtypes or cargo components are responsible for the observed effects, and whether these outcomes are reproducible under standardized or *in vivo* conditions.

### 8.2 Integration of EVs-based strategies in bovine IVF

The potential integration of EVs into bovine ARTs can be conceptually divided into several applications:

- Pre-fertilization phase: EVs derived from reproductive fluids may support oocyte maturation and sperm capacitation, increasing gamete competence. However, functional evidence demonstrating consistent improvements in fertilization rates remains limited.
- Post-fertilization phase: Analysis of EVs cargo in culture media has been proposed as a non-invasive method to assess embryo viability. This approach is promising but currently constrained by variability in biomarker identification and lack of standardized thresholds.
- Embryo culture optimization: Supplementation with EVs from oviductal or uterine origin, may improve developmental outcomes by mimicking physiological signals.
- Therapeutic applications: Engineered EVs have been proposed as delivery vehicles for specific molecular cargoes (e.g., miR-378a-3p or anti-apoptotic miRNAs) to correct deficiencies in embryos cultured under suboptimal conditions.

Overall, while EVs represent a promising avenue for both diagnostic and therapeutic innovation in ARTs, the field is currently characterized by a predominance of associative findings and lack of proof-of-concept studies. Greater emphasis on mechanistic research, rigorous *in vivo* validation, and methodological standardization will be required to substantiate their practical utility. Future studies should prioritize the identification of functionally relevant EVs cargo using validation of candidate biomarkers and therapeutic strategies in physiological models. Addressing these challenges will be critical to transition EVs research from descriptive observations to reliable applications in improving embryo production, fertility, and pregnancy outcomes in both animal models and humans.

## Supplementary material

SUPPLEMENTARY TABLE 1 Summary of extracellular vesicles (EVs) as potential reproductive biomarkers across reproductive compartments. Overview of EV-associated molecular cargo (miRNAs, mRNAs, and proteins) identified in key reproductive compartments and their roles in oocyte maturation, fertilization, and early embryo development. The table also highlights their potential as diagnostic biomarkers reflecting physiological and environmental conditions.

SUPPLEMENTARY TABLE 2 Summarizes methodological variability and MISEV compliance (0–5) across key studies, highlighting generally partial EVs characterization and limited standardization, which may affect reproducibility and interpretation of results. MISEV compliance was qualitatively assessed using a 0–5 scoring system adapted from MISEV2018/2023 recommendations, where higher scores indicate greater methodological rigor in EVs isolation, characterization, and functional validation.

SUPPLEMENTARY TABLE 3 Extracellular vesicle (EV) cargo under different external conditions and functional implications. The table summarizes how external conditions modify EV cargo and their effects on recipient cells. EV responses are context-dependent, with moderate stress promoting protective effects, while chronic or severe stress is associated with altered cargo and impaired developmental outcomes.
