# Supplementary material for: Extracellular vesicles in bovine reproduction: their journey from gametogenesis to pregnancy
Source: Front Cell Dev Biol. 2026 Jun 9;14:1846335. doi: 10.3389/fcell.2026.1846335 (PMC13286908; doi:10.3389/fcell.2026.1846335)
Supplement: Supplementary file 1 [file Table1.docx]

| **Supplementary Table 1. Summary of EVs as potential reproductive biomarkers across reproductive compartments** | | | | |  |
| --- | --- | --- | --- | --- | --- |
| **Structure** | **Main Cargo (miRNAs, Proteins, Lipids)** | **Regulated Pathways / Functions** | **Reproductive Effects** | **Reference** |  |
| **Small follicles (3–6 mm) FF** | miR-323, enriched in EVs, antioxidants | PI3K-Akt, MAPK, WNT, cell cycle | Increases cumulus cell (CC) expansion, alters COC transcriptome, enhances blastocyst rate | Hung et al., 2015, 2017; da Silveira et al., 2017; Andrade et al., 2019 |  |
| **Large follicles (>9 mm) FF** | Higher estradiol content, fewer EVs | Steroidogenesis, reduced proliferation | Stimulates hormone secretion, lower impact on in vitro oocyte maturation | Hung et al., 2017; Andrade et al., 2019; Uzbekova et al., 2020; Ying et al., 2023 |  |
| **Granulosa cells under oxidative stress** | NRF2, antioxidant enzymes in EVs | Oxidative stress defense | Protects recipient cells from oxidative damage | Saeed-Zidane et al., 2017; Gebremedhn et al., 2020 |  |
| **Post-ovulation FF-EVs (in oviduct)** | Mitochondrial proteins, amino acid synthesis factors | Oxidative phosphorylation, thermogenesis | Modulates oviduct epithelium, affects sperm motility & embryo development | Andrade et al., 2019; de Ávila et al., 2020; Tesfaye et al., 2021 |  |
| **Exosomes and microvesicles from FF** | 10 lipids in exosomes, 15 in microvesicles | Lipid signaling pathways | Biomarkers of blastocyst-forming oocytes | da Silveira et al., 2021 |  |
| **Follicular fluid EVs** | miR-31-5p, miR-150, miR-23a | Apoptosis, oxidative stress, follicular development | Promotes oocyte quality and follicle development | da Silveira et al., 2018 |  |
| **Follicular EVs (dominant vs. subordinate follicles)** | miR-21, miR-132, miR-212 | Steroidogenesis, cell proliferation | Influences dominant follicle selection and oocyte maturation | da Silveira et al., 2015 |  |
| **Post-ovulation FF-EVs (in oviduct)** | miR-615 | Targets IGF2 (embryo growth gene) | Embryo development regulator | Mendonça et al., 2015; da Silveira et al., 2017 |  |
| **Exosomes and microvesicles from FF** | miR-323 | Downregulates Eed mRNA in ESCs | Epigenetic control of development | Zhang et al., 2013; da Silveira et al., 2017 |  |
| **Follicular fluid EVs** | PSMA1, PSMA5, PSMD2 (proteasome components) | Protein degradation, immune function | Cell cycle regulation, oxidative stress response | Tanaka, 2009; Uzbekova et al., 2020 |  |
| **Follicular EVs (dominant vs. subordinate follicles)** | miR-181c, miR-193a-3p, miR-29d-3p | Inter-follicular signaling | Reflect body condition and follicular communication | Bastos et al., 2023 |  |
|  | EV-specific lipids (in FF-EVs) | Signal transduction and energy metabolism | Predict oocyte competence to develop into blastocyst | da Silveira et al., 2021 |  |
| **Epididymis**  **Epididymosomes (epithelial cells)** | Tetraspanins (CD9, CD63), integrins, miRNAs (miR-145, miR-143, miR-214 in caput; miR-654, miR-1224 in cauda), proteins: P25b, MIF, AKR1B1, PEBP1, SPAM1, ubiquitin | Epigenetic inheritance, sperm membrane remodeling, protein turnover, chromatin condensation, acquisition of motility | Sperm maturation, motility acquisition, zona pellucida binding | Girouard et al., 2011; Belleannée et al., 2013; Sullivan and Saez, 2013; Sullivan, 2015; Gervasi and Visconti, 2017 |  |
|  | Heat shock proteins, antioxidants, Ca²⁺ regulators, CD9, CD81 | Calcium homeostasis, ROS protection, inhibition of premature capacitation/acrosome reaction | Enhances sperm motility, protects viability, regulates timing of fertilization | Ronquist and Brody, 1985; Girouard et al., 2011; Andrews et al., 2015 |  |
| **Oviductal EVs (OF-EVs)** | CD9, CD81, OVGP1, PMCA4, SPAM1, CATSPER, MYH9, TSG101, HSPA1A, annexins (ANXA1/4/5), miRNAs | Sperm capacitation, acrosome reaction, motility regulation, Ca²⁺ homeostasis, sperm–oocyte fusion, zona pellucida binding | Promotes sperm-oocyte fusion, prevents polyspermy, improves fertilization success | Griffiths et al., 2008; Miyado et al., 2008; Tanigawa et al., 2008; Ohnami et al., 2012; Al-Dossary et al., 2013; Ghersevich et al., 2015; Du et al., 2016; Almiñana et al., 2018; Alcântara-Neto et al., 2020; Franchi et al., 2020; Harris et al., 2020 |  |
| **Post-fertilization (ZP)** | CD9, CD81 | Membrane fusion, EVs transfer to sperm | Essential for sperm-oocyte fusion, CD9-null oocyte rescue via EVs | Miyado et al., 2008; Tanigawa et al., 2008; Ohnami et al., 2012 |  |
| **Oocyte EVs / OF-EVs** | CD9 / CD81 | Sperm-oocyte membrane fusion | Required for fertilization | Miyado et al., 2008; Tanigawa et al., 2008 |  |
| **Oviductal EVs** | PMCA4 | Ca²⁺ regulation, motility control | Prevents premature capacitation, promotes acrosome reaction | Al-Dossary et al., 2013 |  |
|  | OVGP1 | Sperm-ZP binding, CC dispersal | Enhances fertilization | Griffiths et al., 2008b; Almiñana et al., 2017 |  |
|  | miR-375, miR-200 family | Cilia motility, zona pellucida hardening, immune regulation | Promotes sperm capacitation and fertilization success | Almiñana et al., 2017; Lopera-Vasquez et al., 2017; Mazzarella et al., 2021 |  |
| **Epididymosomes / OF-EVs** | SPAM1 | Hyaluronidase activity | Penetration of cumulus matrix | Griffiths et al., 2008b; Girouard et al., 2011 |  |
| **OF-EVs** | CATSPER | Ion channel regulation | Required for sperm hyperactivation | Almiñana et al., 2018 |  |
| **Embryo-derived EVs (days 4–7)** | miR-24-3p, miR-130b-5p, proteins (ICAM1, integrins) | Cell proliferation, adhesion, anti-apoptosis | Promotes embryo development and implantation readiness | Qu et al., 2019; Mazzarella et al., 2021 |  |
| **Embryo**  **(pre-hatching)** | miR-378a-3p, IFNτ, proteins (ISG15, MX1, OAS1Y), cytokines |  | IFNτ induction, anti-inflammatory signaling, bidirectional communication | Maillo et al., 2015; Wydooghe et al., 2017; Talukder et al., 2018; Hamdi et al., 2019; Dissanayake et al., 2021 |  |
|  | bta-miR-26b | Neutrophil activation, cytokine signaling | Enhances embryo survival and endometrial receptivity | Nakamura et al., 2021 |  |
| **Embryo (expanded blastocyst)** | EVs rich in IFNτ, miRNAs, lipoproteins, immunomodulatory proteins (CAPG, AKR1B1) |  | Maternal recognition of pregnancy, luteolysis suppression, ISG activation | Brooks and Spencer, 2015; Burns et al., 2016; Nakamura et al., 2016; Neupane et al., 2017; Kusama et al., 2018; Aguilera et al., 2024 |  |
| **Embryonic EVs (pregnant vs. non-pregnant)** | miRNAs: ↑ bta-miR-126-5p, bta-miR-129 (pregnant); ↑ bta-miR-205, bta-miR-584 (non-pregnant) |  | Embryonic differentiation, immune signaling, embryo quality | Mazzarella et al., 2021 |  |
| **Uterine fluid EVs** | IFNT, ISGs, proteins (osteopontin, galectins) | JAK-STAT, immune modulation, IFN signaling | Prepares endometrium for implantation, signals pregnancy | Burns et al., 2014; Brooks and Spencer, 2015; Kusama et al., 2018 |  |
|  | 195 proteins, 512 mRNAs, 81 miRNAs (e.g., bta-miR-98, miR-499) |  | Trophectoderm proliferation, cell migration, immune tolerance | Burns et al., 2016, 2018; Nakamura et al., 2016; Kusama et al., 2018; Zhao et al., 2019 |  |
| **Cell culture-derived EVs (EECs/BOECs)** | mRNA, lipids, proteins, IFNτ; embryo-derived EVs induce ISG expression in endometrial cells |  | IFNτ response, implantation, differential gene expression | Greening et al., 2016; Sidrat et al., 2020; Dissanayake et al., 2021; Aguilera et al., 2024 |  |
| **Endometrial EVs miRNAs (implantation)** | 172 proteins (VCAM1, annexins), miRNAs | Apoptosis (pre-), adhesion (post-implantation) | Promote trophoblast attachment and uterine receptivity | Kusama et al., 2018 |  |
|  | Fibronectin, FAK-activating proteins | Promotes trophoblast adhesion via FAK signaling |  | Greening et al., 2016 |  |
|  | hsa-miR-200c, hsa-miR-17, hsa-miR-106a | Adhesion, migration, invasion, ECM remodeling |  | Ng et al., 2013; Liang et al., 2017 |  |
|  | Fibronectin, FAK, hsa-miR-200c, hsa-miR-106a | FAK signaling, ECM remodeling | Facilitates implantation via enhanced adhesion | Nakamura et al., 2021 |  |
|  | bta-miR-26b | Immune gene repression (CD40, IER3) | Promotes maternal immune adaptation | Nakamura et al., 2021 |  |
|  | bta-miR-26b | Downregulates PSMC6, CD40, IER3 (immune genes in EECs) |  | Nakamura et al., 2021 |  |
| **Serum EVs (Day 17, embryonic mortality)** | 27 miRNAs (e.g., miR-16, miR-29a) | Apoptosis, angiogenesis, embryonic development | Differentiates pregnancy status and predicts loss | Pohler et al., 2017 |  |
| **Serum EVs (SCNT pregnancies, Day 21)** | Developmental miRNAs | Cell proliferation, differentiation, placental signaling | SCNT-specific pregnancy biomarker | De Bem et al., 2017 |  |
|  | bta-miR-450b, bta-miR-146b, bta-miR-26b, bta-miR-27b | Immune regulation, placental development | Potential diagnostic markers for early gestation | Markkandan et al., 2018 |  |
| **Gestational stage-specific EVs miRNAs** | Day 60: bta-miR-499, miR-16a, miR-20a Day 150: miR-493, miR-127, miR-143 Day 240: miR-122, miR-182, miR-183, miR-200b, miR-200c | Maternal adaptation, fetal development, immune tolerance | Stage-specific support for pregnancy maintenance | Zhao et al., 2019 |  |
