# Supplementary material for: Extracellular vesicles in bovine reproduction: their journey from gametogenesis to pregnancy
Source: Front Cell Dev Biol. 2026 Jun 9;14:1846335. doi: 10.3389/fcell.2026.1846335 (PMC13286908; doi:10.3389/fcell.2026.1846335)
Supplement: Supplementary file 2 [file Table2.docx]

**Supplementary Table 2.** Summarizes methodological variability and MISEV compliance (0–5) across key studies, highlighting generally partial EVs characterization and limited standardization, which may affect reproducibility and interpretation of results.

| **Study** | **EVs source** | **Isolation method** | **Characterization (MISEV criteria)** | **Functional assay** | ***In vivo* relevance** | **Key methodological limitations** | **MISEV compliance (0–5)** |
| --- | --- | --- | --- | --- | --- | --- | --- |
| Aguilera et al., 2024 | Embryo EVs | IVF-derived EVs | RNA-seq | Endometrial cells | Low–moderate | Strong in vitro bias; altered EV cargo | **2** |
| Almiñana et al., 2017 | Oviductal EVs | UC | TEM + partial WB markers | Embryo culture | Moderate | Limited EVs purity confirmation | **3** |
| Almiñana et al., 2021 | Uterine EVs (equine) | UC | Moderate | RNA + lipid analysis | Embryo interaction studies | Lack of single-vesicle validation; heterogeneous populations | 3 |
| Burns et al., 2016 | Uterine EVs (sheep) | UC | Proteomics + miRNA | In vitro uptake | Moderate | No EVs depletion controls | **3** |
| Cañón-Beltrán et al., 2024 | Oviductal EVs | SEC | Functional + pathway analysis | Embryo assays | Moderate |  | **3** |
| da Silveira et al., 2012 (equine) | Follicular fluid | UC | TEM + limited markers | GC uptake assays | Low | No EVs-depletion controls; limited marker validation | **2** |
| da Silveira et al., 2017 | FF-EVs | UC | miRNA profiling only | Embryo culture, miRNA profiling | Target gene prediction | Functional validation absent (no knockdown/overexpression EVs delivery) | **4** |
| De Bem et al., 2017 | Serum EVs | UC | miRNA profiling | Biomarker study | High | Systemic confounders not controlled | **3** |
| Dissanayake et al., 2020 | Embryo culture media | UC | Minimal EVs validation reported | miRNA profiling | Diagnostic potential assay | Lack of EVs enrichment validation; risk of non-vesicular RNA contamination | 1 |
| Fang et al., 2021 | Human IVF culture media | Commercial kit/precipitation | Partial characterization | miRNA profiling | Pregnancy outcome correlation | Precipitation methods likely co-isolate protein aggregates | 1 |
| Franchi et al., 2020 | Oviductal EVs | UC | Partial | Signaling assays | Calcium signaling in sperm | Functional assays in vitro; EV purity not fully defined | 2 |
| Greening et al., 2016 | Human endometrial EVs | UC | Moderate | Protein signaling | Trophoblast adhesion assay | *In vitro*-only; no *in vivo* validation | 3 |
| Gebremedhn et al., 2020 | Follicular fluid EVs | Differential ultracentrifugation | Moderate: NTA + marker proteins reported | miRNA + proteomics | Oocyte competence association | Heterogeneous EVs population; no single-EVs resolution | 3 |
| Hung et al., 2017 | FF-EVs | Differential centrifugation | Size + partial protein profile | CC expansion | Low | Mixed vesicle populations likely | **2** |
| Kusama et al., 2018 | UF-EVs (cow) | UC | Proteomics (limited EVs markers) | EEC assays | Moderate | Possible soluble IFN-τ contamination | **3** |
| Lin et al., 2019 | Embryo culture system | Not clearly specified | Minimal EVs validation | miRNA association | Developmental arrest correlation | EVs origin not confirmed; unclear if RNA is EV-encapsulated | 1 |
| Lv et al., 2018 | Uterine EVs supplementation | UC | Limited EVs validation | Functional embryo assays | Blastocyst formation | EVs cargo not defined; functional attribution uncertain | 2 |
| Leal et al., 2022 | Oviductal/uterine EVs | SEC + UC | Moderate-high: multiple markers reported | Embryo development assays + metabolism genes | Embryo development | Still bulk EVs populations; no subpopulation resolution | **4** |
| Lopera-Vásquez et al., 2016 | Oviductal EVs | UC | TEM + NTA + WB (partial) | IVF embryo development | Moderate | EVs heterogeneity not resolved | **3** |
| Mazzarella et al., 2021 | Oviduct/UF EVs | UC | miRNA sequencing | Observational *in vivo* | High | Correlative design only | **3** |
| Mazzarella et al., 2025 | Oviduct/uterine EVs | UC + *ex vivo* model | Proteomics + comparative validation | *In vivo* + explant | High | *Ex vivo* not fully physiological | **4** |
| Mellisho et al., 2017 | Bovine embryo culture media | UC (reported) | Limited: TEM + NTA (partially reported) | Not fully defined | Embryo development assay | Possible co-isolation of non-EVs particles; limited marker validation (CD63/CD9 not consistently reported) | 2 |
| Mellisho et al., 2019 | Bovine blastocyst culture media | Differential centrifugation | Partial: size and concentration reported | No deep proteomic/miRNA validation | Embryo viability correlation | Correlative design; EVs purity uncertain; no density gradient purification | 2 |
| Melo-Baez et al., 2020 | Embryo culture media | Precipitation-based method | Limited characterization | miRNA sequencing | Biomarker identification | High risk of protein/lipoprotein contamination; no EVs-specific markers | 1 |
| Mendonça et al., 2015 | Follicular fluid EVs | UC | Limited marker validation | Proteomics | Functional inference | No functional EVs transfer validation | 2 |
| Nakamura et al., 2016 | UF-EVs | UC | WB + qPCR | EEC signaling assays | Moderate | Cytokine co-isolation likely | **3** |
| Pavani et al., 2022 | Blastocyst EVs | EVs isolation (mixed methods) | miRNA + functional assays | Gene knockdown | Moderate | Mostly *in vitro* validation | **4** |
| Sullivan, 2015 | Epididymosomes | Biological isolation (fluid fractionation) | High EVs definition accepted in field | Proteome + miRNA | Sperm maturation studies | Strong biological relevance but limited standardized EVs isolation reporting | 3 |
| Zhao et al., 2019 | Serum EVs | UC | miRNA profiling | Biomarker analysis | High | Correlative only | **3** |
| Zhang et al., 2019 | Embryo culture media | Not specified | Limited | miRNA profiling | Correlative study | No EVs purification standards described | 1 |

MISEV compliance was qualitatively assessed using a 0–5 scoring system adapted from MISEV2018/2023 recommendations, where higher scores indicate greater methodological rigor in EVs isolation, characterization, and functional validation.
