# Supplementary material for: Extracellular vesicles in bovine reproduction: their journey from gametogenesis to pregnancy
Source: Front Cell Dev Biol. 2026 Jun 9;14:1846335. doi: 10.3389/fcell.2026.1846335 (PMC13286908; doi:10.3389/fcell.2026.1846335)
Supplement: Supplementary file 3 [file Table3.docx]

**Supplementary Table 3. Extracellular vesicle (EV) cargo under different external conditions and their functional implications.**

| **External condition** | **Changes in EVs cargo** | **Effects on recipient cells / oocytes** | **Functional interpretation** | **Evidence level** | **Reference** |
| --- | --- | --- | --- | --- | --- |
| **Oxidative stress** | ↑ Nrf2, ↑ antioxidant enzymes | Protects cells from reactive oxygen species (ROS); reduces oxidative stress | Beneficial adaptation | Causal (in vitro) | Gebremedhn et al., 2020 |
| **Heat stress** | ↑ Heat shock proteins (HSPs) in EVs | “Bystander effect” → recipient cells become more thermotolerant, ↓ apoptosis, ↑ embryo development | Protective adaptation | Causal (in vitro); Correlative (in vivo) | Bewicke-Copley et al., 2017; Rodrigues et al., 2019 |
| **Metabolic stress** | ↓ miR-132, ↓ miR-34b/c, ↓ miR-449a (in NEB cows); ↑ bta-miR-489 (in moderate reserves) | Associated with reduced oocyte competence; altered EV-mediated communication between compartments | Potentially detrimental | Correlative | Hailay et al., 2019; Bastos et al., 2023 |
| **Early follicular stage** | ↑ Total EV concentration, ↑ miRNAs, specific proteins | Enhanced cumulus cell expansion; likely support oocyte maturation | Pro-developmental role | Correlative | Hung et al., 2015; Gebremedhn et al., 2020; Uzbekova et al., 2020 |
| **Late follicular stage** | ↓ EV concentration; altered miRNA/protein profiles | Reduced cumulus expansion; may reflect preovulatory changes or inhibitory signaling | Fine-tuning or regulatory shift | Correlative | Hung et al., 2015; Uzbekova et al., 2020 |
| **Lipid profile in FF-EVs** | 514 lipids identified; 10 specific to exosomes, 15 to microvesicles in competent oocytes | Lipids associated with oocytes that developed into blastocysts; potential biomarkers | Linked to oocyte competence | Correlative | da Silveira et al., 2021 |

**NEB:** Negative Energy Balance; **↑ / ↓:** Increase / Decrease; **FF-EVs:** Extracellular vesicles from follicular fluid; **Evidence Level:** Indicates whether data is correlational (observational) or supported by experimental (causal) evidence.
